# N-Terminally Myristoylated Feline Foamy Virus Gag Allows Env-Independent Budding of Sub-Viral Particles

**DOI:** 10.3390/v3112223

**Published:** 2011-11-14

**Authors:** Yang Liu, Yong-Boum Kim, Martin Löchelt

**Affiliations:** Department of Genome Modifications and Carcinogenesis, Research Program Infection and Cancer, German Cancer Research Center (Deutsches Krebsforschungszentrum, DKFZ), Im Neuenheimer Feld 242, 69120 Heidelberg, Germany; E-Mails: y.liu@dkfz.de (Y.L.); kim@nmr.uni-frankfurt.de (Y.-B.K.)

**Keywords:** feline foamy virus, retrovirus, assembly, budding, release, sub-viral particles, myristoylation

## Abstract

Foamy viruses (FVs) are distinct retroviruses classified as *Spumaretrovirinae* in contrast to the other retroviruses, the *Orthoretrovirinae*. As a unique feature of FVs, Gag is not sufficient for sub-viral particle (SVP) release. In primate and feline FVs (PFV and FFV), particle budding completely depends on the cognate FV Env glycoproteins. It was recently shown that an artificially added N-terminal Gag myristoylation signal (myr-signal) overcomes this restriction in PFV inducing an *Orthoretrovirus*-like budding phenotype. Here we show that engineered, heterologous N-terminal myr-signals also induce budding of the distantly related FFV Gag. The budding efficiency depends on the myr-signal and its location relative to the N-terminus of Gag. When the first nine amino acid residues of FFV Gag were replaced by known myr-signals, the budding efficiency as determined by the detection of extracellular SVPs was low. In contrast, adding myr-signals to the intact N-terminus of FFV Gag resulted in a more efficient SVP release. Importantly, budding of myr-Gag proteins was sensitive towards inhibition of cellular N-myristoyltransferases. As expected, the addition or insertion of myr-signals that allowed Env-independent budding of FFV SVPs also retargeted Gag to plasma membrane-proximal sites and other intracellular membrane compartments. The data confirm that membrane-targeted FV Gag has the capacity of SVP formation.

## 1. Introduction

Virus particle assembly and their subsequent or concomitant release from the host cell are important steps during viral replication clearly contributing to their replication competence and infectivity [1]. Thus, the associated processes are subject to host-mediated restriction, but are also of critical importance for translational approaches exploiting viruses as gene delivery or vaccine antigen expression and presentation vehicles [2]. In this context, requirements important for virus transmission and survival in the host must not automatically fit those relevant for gene transfer or vaccine antigen expression. Thus, molecular dissection of these processes is not only aimed at increasing our overall understanding of the molecular biology of the corresponding viruses, but may also help to exploit or even optimize the mechanisms identified [3].

Foamy viruses (FVs), also designated Spumaretroviruses, are distinct retroviruses with many features of their molecular biology and replication strategy clearly different from the canonical Orthoretroviruses like HIV, MLV and HTLV. For example, FV capsid assembly occurs at the microtubule organizing center (MTOC) while budding through different cellular membranes and subsequent release of intact virus particles absolutely depends on the presence of FV Env glycoproteins. In addition, virus maturation appears to be temporarily delayed and is accompanied with only partial Gag processing which occurs mainly at a C-terminal site (see below and reviewed in [4–6]). The unique features of FVs may be related to the long FV-host co-evolution, a comparably unconventional gene expression strategy and unique features of most of the FV proteins [7,8].

Some of these characteristics include the apparent apathogenicity and persistent replication in their natural hosts, the Gag-independent Pol expression and their high genetic stability which makes them interesting and promising candidates for different applications in targeted gene delivery and vaccine antigen expression and presentation [9,10].

Many of the distinguishing FV-specific features have been explored using the prototype FV (PFV), formerly called human FV which represents the end-product of the zoonotic transmission of a chimpanzee FV to a human being [11]. Since PFV has crossed species barriers, had been propagated for extended periods in various human and non-human cells and suffered gross genetic deletions in the regulatory long terminal repeats (LTR) [12], it is open as to whether some or all PFV-specific features are also shared by distantly related FVs, for example feline FV (FFV) [10].

Among the unique FV-specific features is the strict Env surface protein-dependence of particle budding mediated by a highly specific interaction of N-terminal Gag and Env sequences [13–15]. In addition and in contrast to most other retroviral Gag proteins, FV Gag proteins do not encode N-terminal myristoylation (myr) signals [16] and in fact, FV capsid assembly occurs independent of cellular membranes in the proximity of the MTOC similar to D-type retroviruses like Mason-Pfizer Monkey Virus [17–19]. Eastman and Linial showed in the context of a replication-competent PFV DNA clone that the Gag R58A mutant with abolished particle assembly and MTOC targeting could be rescued for particle assembly and release by Gag myristoylation [17].

Recent studies showed that the Env-dependency of FV budding is not absolute: prototype FV (PFV) Gag engineered to carry different N-terminal myr-signals was shown in two independent studies to induce the release of Gag-only SVPs [20,21]. However, a myristoylated Gag expressed from a proviral PFV DNA did not support viral infectivity and marker gene transfer [20].

The N-terminal myristoylation of cellular and viral proteins is a routine mechanism to target and anchor proteins via the strongly hydrophobic myristic acid to diverse membrane compartments, often an essential prerequisite for proper function [22,23]. Myr-signals are encoded by N-terminal amino acids of membrane-targeted proteins and are recognized by cellular N-myristoyltransferases (NMTs) for the addition of myristate to the (sub)terminal glycine residue of the target protein [22,23].

Here we studied whether FFV Gag also has the capacity to bud from cells simply by adding N-terminal myr-signals. The addition of N-terminal myr-signals and, to a lesser degree, the substitution of FFV Gag sequences by myr-signals induced SVP budding. Importantly, the release was abrogated specifically by the inhibition of cellular NMTs.

## 2. Results

### 2.1. FFV Gag Myr-Substitution Clones and Particle Budding

**Figure 1.**
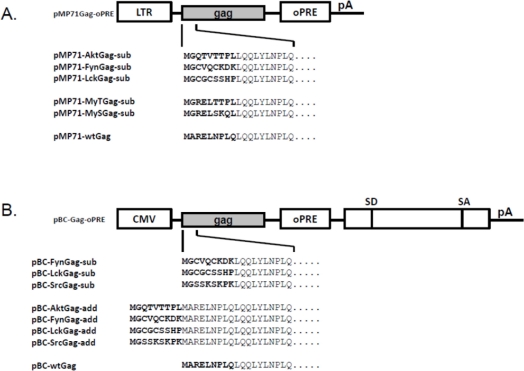
Schematic structure of the different feline foamy viruses (FFV) Gag expression clones used. (**A**) In the pMP71Gag-oPRE-based FFV myr-Gag expression clones, amino acids 2 to 9 of FFV Gag were exchanged by sequences from the human cellular proteins Akt, Fyn and Lck. In addition, the FFV Gag sequence was adapted to myr-signals of the serine- (MyS) and the threonine-type (MyT) [22]. (**B**) In the pBC-Gag-oPRE FFV Gag expression clones, myr-signals derived from human cellular proteins Akt, Fyn, Lck and Src were added (add) or inserted (sub) N-terminally to the FFV Gag sequence. Schematically, the positions of the LTR and CMV-IE promoter, the optimized post-transcriptional-regulatory element (oPRE), a non-coding heterologous sequence providing splice-donor (SD) and splice-acceptor (SA) sites as well as the poly-A-addition site (pA) are indicated, for details, see reference [25].

In order to determine whether the Env-independent budding of myristoylated Gag proteins is a shared feature of FVs and not only a specific characteristic of PFV [20,21], we engineered FFV Gag proteins in such a way that the first nine amino acid residues were replaced (substituted) by known myr-signals of other proteins known to be membrane targeted via this N-terminal protein modification (Figure 1A). The myr-signals were derived from the cellular membrane-associated Akt, Fyn and Lck proteins. These myristoylation-substitution (myr-sub) clones are similar to those used by Life *et al.* [20] for PFV. In addition, we aimed at changing the original FFV Gag sequence with only minimal changes to T- or S-type-like myr-signals with either a serine or threonine at position six starting at the N-terminal Met residue [22]. The mutations were introduced into an FFV Gag expression construct based on the retroviral vector genome pMP-71 [24,25].

**Figure 2.**
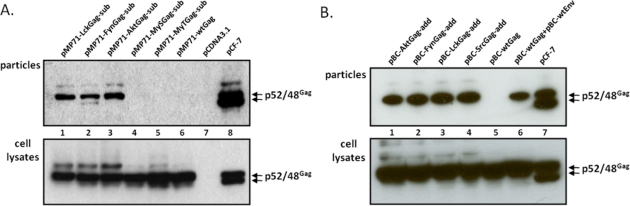
FFV myr-Gag is released as sub-viral particles (SVPs). Proteins of cell lysates and virus particle preparations (as labeled) were harvested 2 days after transfection and assayed for FFV Gag by immunoblotting. (**A**) 293T cells were transfected with FFV myr-sub Gag expression clones pMP71-LckGag-sub, pMP71-FynGag-sub, pMP71-AktGagsub, pMP71-MySGag-sub, pMP71-MyTGag-sub (lane 1 to 5), the unmodified parental wt FFV Gag expression clone pMP71-wtGag (lane 6), the full-length FFV genome pCF-7 (lane 8) and control plasmid pcDNA3.1 (lane 7). (**B**) 293T cells were transfected with FFV myr-add Gag expression clones pBC-AktGag-add, pBC-FynGag-add, pBC-LckGag-add, pBC-SrcGag-add (lane 1 to 4), the unmodified parental wt FFV Gag expression clone pBC-wtGag (lane 5) and the full-length FFV genome pCF-7 (lane 7). In parallel, 293T cells were co-transfected with wt FFV Gag expression clone pBC-wtGag and wt FFV Env expression clone pBC-wtEnv (lane 6). The positions of the FFV p52 and p48 Gag proteins are marked.

Upon transfection of 293T cells with the different FFV myr-sub Gag-encoding plasmids, the unmodified parental WT FFV Gag expression clone, the full-length and replication-competent FFV genome pCF-7 and a negative control (pcDNA), cell-associated antigens (Figure 2A, bottom) and the release of SVPs (top) were analyzed two days later. WT and modified FFV Gag proteins of 52 kDa were expressed in transfected cells at comparable levels, however only the Gag protein of the full-length infectious FFV proviral DNA clone pCF-7 was processed to the additional 48 kDa form. Budding of FFV SVPs was only detectable upon insertion of a canonical myr-signal (Figure 1A) while WT Gag or Gag variants with engineered S- and T-type-like myr-signals [22] did not bud from cells. Budding of intact FFV particles as determined by densitometry of the image was about four-fold more efficient than myr-Gag SVP release and was associated with partial Gag processing due to the fact that all viral proteins including Pol were expressed in the cells. The nature of the FFV Gag-specific bands above the p52 FFV Gag precursor is currently not known. They are present to different degrees in samples from WT and myr-Gag-expressing cells (see discussion).

In order to increase overall protein expression [25], some of these mutations were subcloned into the pBC12-background which provides a downstream splice acceptor and splice donor pair together with an inserted optimized woodchuck hepatitis virus post-transcriptional regulatory element (oPRE, Figure 1B) [25]. In addition, the myr-signal of the Src protein was also engineered into the N-terminus of FFV Gag. Except for the expected increase in overall protein expression, the pBC12-based clones were undistinguishable from the pMP-71-based expression clones (data not shown).

### 2.2. FFV Gag Myr-Addition Clones and Particle Budding

Subsequently, the functional, canonic myr-signals were also directly added to the FFV Gag N-terminus (myr-add clones) in the pBC12-background, a strategy aimed at maintaining the N-terminal FFV Gag sequences fully intact (Figure 1B). The N-terminal addition of the myr-signal had been also employed by Zhadina *et al.* [21]. Again, these constructs were tested for gene expression and budding competence in parallel to WT Gag expression construct and the FFV proviral genome. In addition, 293T cells were also co-transfected with WT FFV Gag and Env expression constructs. Two days after transfection, similar amounts of WT and myr-add Gag were expressed (Figure 2B, bottom panel), however, only FFV Gag proteins carrying myr-signals were budding competent. The unmodified Gag was only released from the cells in the form of particulate SVPs that can be sedimented through a 20% sucrose cushion when FFV Env was co-expressed (Figure 2B, top panel). When comparing FFV virus release and Gag SVP budding by densitometry of an autoradiogram where the signals were in a linear range, SVP particle release was about 40 to 50 % of that of intact and infectious FFV.

When the pBC12-based myr-add and myr-sub Gag expression clones were directly compared, the myr-add clones were consistently (three- to nine-fold) more efficient in directing SVP budding compared to the myr-sub clones (data not shown).

### 2.3. The Budding Capacity of FFV Myr-Gag Proteins Is Myristoylation-Dependent

**Figure 3.**
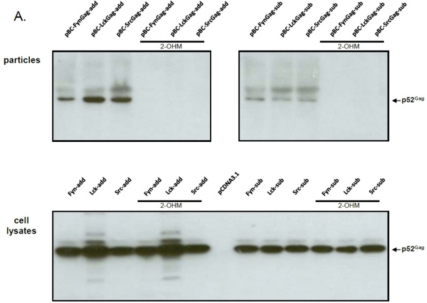

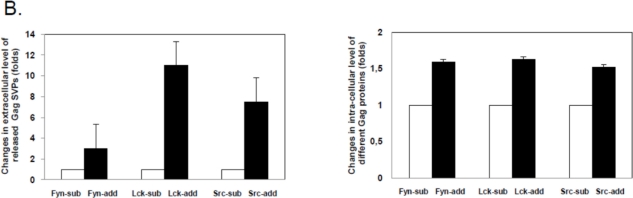
FFV myr-Gag SVP budding is abrogated by the N-myristoyltransferases (NMT) inhibitor 2-OHM. (**A**) 293T cells were transfected with myr-Gag expression clones (as given above the figures). The NMT inhibitor DL-2-hydroxymyristic acid (2-OHM) was added after the medium change at the end of transfection (about 10 hours after DNA addition) and left on the cells for at least 30 hours. Proteins of cell lysates and particle preparations were harvested and assayed by immunoblotting for FFV Gag. The positions of the FFV p52 Gag protein and β-actin are marked. (**B**) Densitometric quantification of the relative expression levels and budding capacities of the different FFV myr-Gag proteins in the absence of 2-OHM shows similar intracellular levels of the different FFV Gag myr-sub and myr-add clones (white and black bars, resp.) while the budding capacity of the different FFV Gag myr-add proteins was significantly higher than that of the myr-sub Gag proteins.

In order to determine whether budding was dependent on the activity of the host-encoded, cellular NMT that mediates the N-terminal modification of FFV Gag molecules carrying a myr-signal, NMT inhibitor studies were performed. As described in the Experimental Section, the NMT inhibitor DL-2-hydroxymyristic acid (2-OHM) was added after the medium change at the end of transfection (about 10 hours after DNA addition) and left on the cells for at least 30 hours. No obvious toxicity of 2-OHM was detectable during this time. While Gag expression from all myr-sub clones was slightly lower, the overall Gag expression from either the myr-add and myr-sub clones was unaffected by the presence of 2-OHM (Figure 3A). In line with our findings and substantially corroborating previous studies [20,21], budding of FFV myr-Gag particles was completely abrogated in the presence of the NMT inhibitor 2-OHM. In the absence of the inhibitor, budding occurred consistently. Again, densitometry of cell-associated *versus* released myr-Gag proteins confirmed an enhanced budding capacity of the myr-add clones compared to the myr-sub proteins (Figure 3B). 2-OHM did not affect the expression, stability and budding-incompetence of WT FFV Gag (data not shown).

### 2.5. N-Terminal Deletions in Myr-Gag Proteins Interfere with Budding

**Figure 4.**
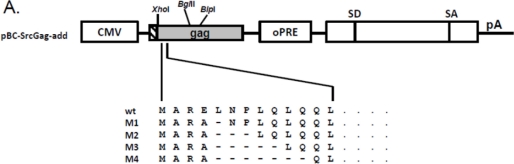

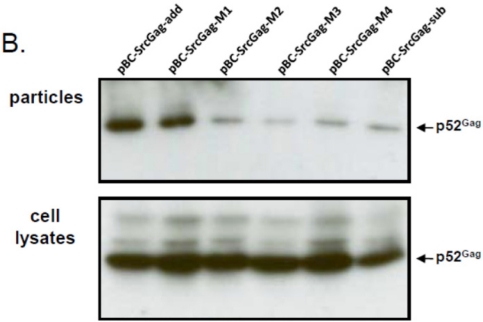
N-terminal deletions reduce the budding capacity of FFV myr-add Gag. (**A**) Schematic structure of the FFV SrcGag-add deletion mutants. Genetic features of the plasmids are labeled as in Figure 1 and the positions of the *Xho*I, *Bgl*II and *Blp*I restriction enzymes used for cloning are marked. (**B**) SVP production by 293T cells transfected with different FFV Src-add Gag deletion mutants, the parental Src-add Gag clone and the Src-sub clone were used as controls (as given above the image). Gag expression (bottom panel) and SVP release (top panel) were analyzed by immunoblotting using the corresponding samples taken two days after transfection. The position of the FFV p52 Gagproteins is marked.

To analyze whether the N-terminal deletions of FFV Gag sequences may be in fact responsible for the reduced budding capacity of the myr-sub Gag clones, increasing amino acid deletions were introduced into the FFV Gag Src-myr-add clone as displayed in Figure 4A. All mutant and control Gag proteins were expressed at comparable levels 2 days after transfection into 293T cells (Figure 4B, bottom panel). The budding efficiency of mutant M1 (one deletion and one amino acid exchange) was similar to that of the full-length Src-add Gag (Figure 4B, top panel). In clear contrast, consecutive two-amino acid deletions displayed a strongly compromised budding phenotype similar to the Src-sub Gag clone used as control. This finding substantiates the hypothesis that N-terminal sequences are required for full biological activity of Gag. In addition, when the M1 to M4 deletions were analyzed in the context of the WT FFV genome encoding unmyristoylated Gag, only the M1 mutant displayed WT infectivity (data not shown).

### 2.6. FFV Myr-Gag Proteins Are Relocalized to the Plasma Membrane in Transfected Cells

**Figure 5.**
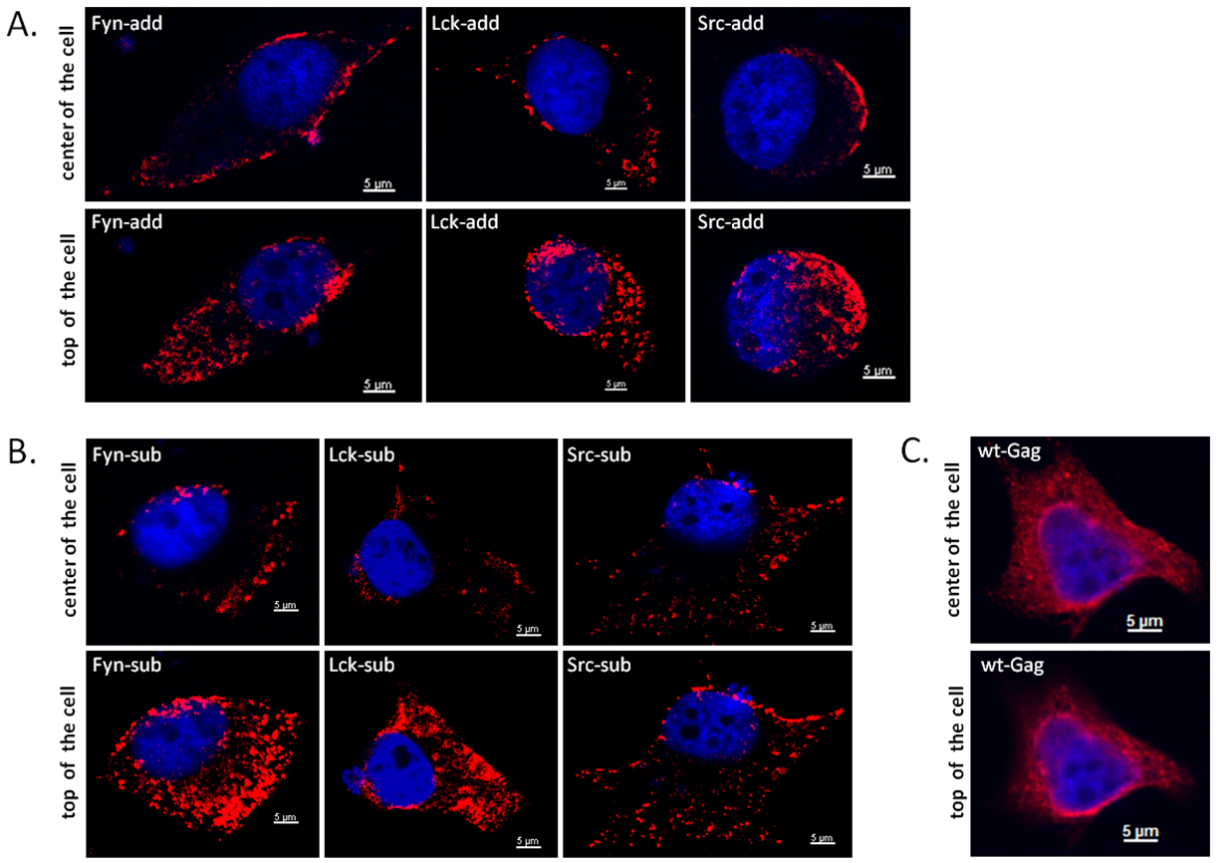
Subcellular distribution of WT and myr-Gag proteins as determined by indirect immunofluorescence (IIF) confocal laser microscopy of transfected and fixed HeLa cells. HeLa cells were transfected with plasmids directing expression of myr-add (**A**), myr-sub (**B**), and WT, unmodified Gag (**C**). Cells were fixed with para-formaldehyde and reacted with a guinea pig antiserum against the FFV Gag capsid domain (red staining) while nuclei were counter-stained with DAPI (blue staining) as described in the Experimental Section. Composite z stacks of two confocal sections (out of 25) are shown. Single confocal sections were acquired at the center of the cells (upper panels) and at the apical, top part of the cells (lower panels).

Next, the subcellular localization of WT and myr-Gag proteins was determined. HeLa cells were used for these confocal microscopy studies since 293T cells used in the other experiments are not well-suited for such experiments due to their low cytoplasmatic content. One day after DNA transfection of the HeLa cells with WT and mutant Gag clones, the cells were fixed with para-formaldehyde and subjected to indirect immunofluorescence (IIF) using an FFV CA-specific antiserum as described in the Experimental Section. Representative confocal microscopy images of central and apical sections of Gag-expressing cells are displayed in Figure 5. While WT Gag displays a complex subcellular localization with fine-granular cytoplasmic staining associated with a prominent peri-nuclear staining (panels C), a clear co-localization of any of the myr-add and myr-sub mutants (panels A and B, resp.) with the nuclear periphery is not detectable. In sharp contrast and anticipated upon addition on the membrane-targeting myr-signals to FFV Gag, a strong and consistent association to the plasma membrane is clearly detectable for all myr-Gag proteins. In addition, cytoplasmatic myr-Gag is detectable in a coarse-granular pattern which likely corresponds to a co-localization of myr-Gag proteins with intracellular membrane compartments.

## 3. Experimental Section

### 3.1. Cell Culture and DNA Transfection

293T and HeLa cells were grown in DMEM (Sigma-Aldrich, Germany) supplemented with 10% fetal calf serum (PAN Biotech, Aidenbach, Germany) and 1% penicillin-streptomycin (Sigma-Aldrich, Taufkirchen, Germany). Transfection of 293T cells with plasmids DNA was performed by calcium co-precipitation of sub-confluent 293T cells in 20 cm^2^ dishes as described previously [26]. Cells and cell culture supernatants were harvested 40 to 44 hours after transfection. In order to inhibit cellular NMTs, 2-hydroxymyristic acid (2-OHM; Sigma-Aldrich) was added to the medium at a final concentration of 0.1 mM from 10 to about 40 hours post-transfection.

### 3.2. Purification of FFV Particles and SVPs

Each 5 mL of cell culture supernatants of transfected 293T cells were cleared of cellular debris by centrifugation for 10 min at 405 g. Subsequently, FFV particles or SVPs were pelleted through a 2 mL cushion of 20% sucrose in PBS (w/v) by ultracentrifugation in a SW41Ti rotor (Beckman Coulter, Krefeld, Germany) for 2 hours at 100,000 g and 4 °C [27]. The non-visible pellet devoid of medium and sucrose was re-suspended in 50 μL of 1% SDS in PBS, containing Complete Protease Inhibitor Cocktail (Roche, Mannheim, Germany) and stored at −20 °C before immunoblot analyses.

### 3.3. Molecular Cloning of FFV Gag Derivatives

The murine leukemia virus vector-based WT FFV Gag expression plasmids pMP71Gag-oPRE and the CMV-IE promoter-based Gag expression clone pBC-Gag-oPRE utilizing an optimized post-transcriptional-regulatory element (oPRE) from the woodchuck hepatitis virus have been described previously [25]. The infectious FFV molecular clone pCF-7 [28] served as control for particle release.

In order to incorporate myr-signals into the N-terminus of FFV Gag, the FFV Gag myr-sub clones pMP71-AktGag-sub, pMP71-FynGag-sub, pMP71-LckGag-sub, pMP71-MyTGag-sub and pMP71-MySGag-sub, were constructed as follows: myr-Gag expression plasmids were derived from plasmid pMP71Gag-oPRE in which the N-terminal nine amino acids of FFV Gag were exchanged by the nine amino acid-long myr-signals of the Akt, Fyn and Lck proteins. In addition, the hypothetical myr-signals of the serine- and threonine-type (MyS and MyT, resp.) were used to generate two additional myr-sub Gag mutants. FFV Gag was amplified using the reverse primer R-Gag985 and each forward primers F-Akt, F-Fyn, F-Lck, F-MyT, or F-MyS (Table 1). The forward primers anneal to the N-terminus of FFV Gag and provide the myr-signal and an *Age*I restriction site at the 5′-end. The reverse primer R-Gag985 anneals at residue 984 bp of FFV Gag and encloses a unique *Blp*I restriction site (Table 1). Phusion high fidelity polymerase was used for high-fidelity amplification as recommended by the supplier (Roche, Mannheim, Germany). PCRs were performed in a Mastercycler (Eppendorf, Hamburg, Germany) in 50μL volumes at 35 cycles of 95 °C, 15 sec denaturation, 54 °C, 30 s annealing and 72 °C, 60 s elongation. The *Age*I- and *Blp*I-digested pMP71Gag-oPRE and the correspondingly digested PCR products were then ligated and recombinant clones were identified by standard procedures.

The pMP71-based myr-gag sequences of pMP71-LckGag-sub and pMP71-FynGag-sub were sub-cloned into the pBC-Gag-oPRE background by exchange of the corresponding *Age*I to *Blp*I fragment to generate pBC-LckGag-sub and pBC-FynGag-sub. Independently, FFV Gag was amplified using the forward primers F-Src and reverse primer R-Gag985 as described above (Table 1). The *Age*I-and *Blp*I-digested pBC-Gag-oPRE and PCR products were then ligated to generate pBC-SrcGag-sub.

To add functional myr-signals to the N-terminus of FFV Gag, the myr-add clones pBC-AktGag-add, pBC-FynGag-add, pBC-LckGag-add and pBC-SrcGag-add were constructed as follows: The Akt, Fyn, Lck and Src myr-signals were added to the N-terminus of FFV Gag to avoid deletion of critical FFV Gag residues or a possible misfolding of the N-terminus of Gag and the subsequent loss of function. For this purpose, oligonucleotides 1, 3, 5 and 7, which contain the Akt, Fyn, Lck and Src myr-signals were annealed to their complementary oligonucleotides 2, 4, 6 and 8, resp. (Tables 2 and 3). The annealed oligonucleotides are flanked by *Age*I and *Xho*I overhang ends and were so directly ligated into *Age*I- and *Xho*I-digested pBC-Gag-oPRE to generate the corresponding myr-add Gag mutants.

PCR-mediated mutagenesis was used to introduce different small N-terminal Gag deletions into pCF-7. For this purpose, PCR sense primers M1 to M4 and antisense primer AS (Table 4) were used as described above. The amplicons were digested with *Xho*I and *Blp*I and inserted into the correspondingly digested pCF-7, the schematic structure of the mutants and the restriction sites used are depicted in Figure 4A. The new constructs are designated pCF-7-M1, -M2, -M3 and -M4, resp. To investigate the role of added myr-signals on the M1 to M4 Gag mutants, these deletions were excised with *Xho*I and *Bgl*II and inserted into the correspondingly digested pBC-SrcGag-add clone to generate pBC-SrcGag-M1, pBC-SrcGag-M2, pBC-SrcGag-M3, pBC-SrcGag-M4.

The introduced mutations in all engineered plasmid DNA constructs generated in this study were confirmed by DNA sequencing.

### 3.4. Protein Detection by Immunoblotting

Transfected cells were harvested by lysis in 1% SDS. Immunoblotting was performed as previously described [29]. Expression of Gag was visualized using guinea pig antiserum against the capsid CA domain of FFV Gag [30] and with HRP-coupled mouse antibody against β-actin (Sigma-Aldrich, Germany). The resulting images were quantified by densitometry using the ImageJ software [31].

### 3.5. IIF and Confocal Laser Microscopy

HeLa cells were seeded on coverslips placed in 12 well-plates. Transfection of HeLa cells with plasmids DNA was performed with Lipofectamine^TM^ 2000 according to the manufacturer’s instructions (Invitrogen, Karlsruhe, Germany). In brief, 1.5 μg of FFV Gag expression constructs plus 0.2 μg of an EGFP expression plasmid were co-transfected. About one day after transfection, cells were fixed in 3% paraformaldehyde (10 min), permeabilized with 0.1% Triton-X 100 (5 min), and blocked with 3% BSA (in PBS; 30 min). The fixed cells were then incubated with guinea pig antiserum against the capsid domain of FFV Gag (1:2,000) for one hour. The primary antibody was detected with goat-α-guinea pig Alexa Fluor 568 (1:16,000) for 45 min and nuclei were stained with Hoechst 33342 (1:1,000). After three final PBS washes, the samples were mounted on microscope slides (Thermo Scientific, Braunschweig, Germany).

Laser scanning confocal microscopy was performed on a Zeiss LSM700 confocal microscope. Laser beams with 364- and 555-nm excitation wavelengths were used for Hoechst and Gag imaging, resp. Z stacks of typically 25 0.4-mm slices were taken and single confocal sections and z-stack images were processed using AxioVision software [32].

## 4. Discussion and Conclusions

In the current study we demonstrate that myristoylation of FV Gag proteins in fact allows Env-independent budding of FV Gag-only SVPs. The data presented here for FFV confirm and extend previous studies for the distantly related PFV. Most importantly, we demonstrate by the use of a specific inhibitor of cellular NMTs that the Env-independent budding of FV Gag proteins carrying N-terminal myr-signals is in fact myristoylation-dependent and not simply due to the addition or insertion of the corresponding heterologous protein motifs. However, it is currently unclear whether slower-migrating Gag protein bands, detectable predominantly in cell extracts and released particles from myr-Gag proteins, are due to Gag myristoylation or other budding-associated Gag protein modifications. In addition, the extent of FV Gag myristoylation required to induce SVP budding is currently unknown. The Env-independent budding of FFV myr-Gag is highly specific since we never detected WT, unmyristoylated Gag proteins in the particulate extracellular fraction upon sedimentation through a sucrose cushion and since transmission electron microscopy studies revealed plasma membrane-derived budding structures of myr-Gag proteins alone [29].

Previous studies on the distantly related PFV used two different methods for PFV Gag myristoylation [20,21]. Importantly, we used and compared both strategies by adding the myr signal to the N-terminus of Gag, or by substituting N-terminal FFV Gag sequences by the myr-signals in a way similar to the studies on PFV Gag by Life *et al.* and Zhadina *et al.*, respectively [20,21]. Although the corresponding FFV myr-add and myr-sub Gag proteins displayed similar intracellular steady-state levels and subcellular localization pattern within the transfected cells (see Figures 3 and 5), the Env-independent release of particles with fully intact N-terminal FFV Gag sequences (myr-add clones, Figure 3) was reproducibly more efficient than that of particles with deletion of the nine amino acid N-terminal FFV Gag sequence due to their replacement by the myr-signals (myr-sub clones, Figure 3). Importantly, it was not possible by minimal mutagenesis to subvert the authentic Gag N-terminus to a myr-signal. It appears that all authentic cell-derived myr-signals tested in this study worked with similar efficiency when studied in the context of the FFV Gag protein. However, in the experimental system employed here, the intactness of Gag and especially of its N-terminus is the critical factor for the particle assembly and budding efficiency.

The importance of these N-terminal Gag sequences for particle release upon myristoylation is confirmed by the data shown in Figure 4. In addition, these N-terminal Gag sequences are also of importance for WT, non-myristoylated Gag since FFV infectivity when assayed in the context of a full-length and replication-competent FFV genome is strongly reduced by the corresponding N-terminal Gag deletions [33]. Since myr-add and myr-sub Gag proteins display similar stability and sub-cellular localization within the cells, the detrimental deletions in the myr-sub clones and the M2 to M4 deletions are not likely to affect overall protein stability or to induce bulk protein sequestration or aggregation. Recent studies on FV Gag showed that N-terminal Gag sequences/motifs are required for proper cytosolic capsid formation and interaction with a highly conserved N-terminal Env domain required for Env-dependent wt Gag budding [14,15,17–19,34]. In contrast, this region surprisingly functions as a retroviral late domain, since there is no obvious similarity to corresponding sequences of other retroviruses and since late domains have been mapped to other parts of FV Gag proteins [14,15,17–19,34]. Additional studies on these and related issues will be required to gain a comprehensive understanding of the processes of FV particle assembly and release.

## Figures and Tables

**Table 1.** Oligonucleotides used for PCR-mediated myr-sub mutagenesis.

**Primer Name**	**Sequence ^a^**
F-Fyn	5′-CGAGACCGGTCCGCCATGGGCTGCGTGCAGTGCAAGGACAAGTTACAGCAACTGTATATA-3′
F-Lck	5′-CGAGACCGGTCCGCCATGGGCTGCGGCTGCAGCAGCCACCCCTTACAGCAACTGTATATA-3′
F-Akt	5′-CGAGACCGGTCCGCCATGGGCCAGACCGTGACCACCCCCCTGTTACAGCAACTGTATATA-3′
F-MyT	5′-CGAGACCGGTCCGCCATGGGCCGGGAACTGAGCAAGCTGCAATTACAGCAACTGTAT-3′
F-MyS	5′-CGAGACCGGTCCGCCATGGGCTGCGGCTGCAGCAGCCACCCCTTACAGCAACTGTATATA-3′
F-Src	5′-GCTTACCGGTCCGCCATGGGCAGCAGCAAGAGCAAGCCCAAGTTACAGCAACTGTATATA-3′
R-Gag985	5′-TGTATAGATTGTTAATGACCCTGG-3′

a *Age*I restriction site is underlined.

**Table 2.** Oligonucleotides used for myr-add mutagenesis.

**Primer Name**	**Sequence ^a^**
1	5′-CCGGTACCGCCATGGGCCAGACCGTGACCACCCCCCTGATGGC-3′
2	5′-**TCGA**GCCATCAGGGGGGTGGTCACGGTCTGGCCCATGGCGGTA-3′
3	5′-CCGGTACCGCCATGGGCTGCGTGCAGTGCAAGGACAAGATGGC-3′
4	5′-**TCGA**GCCATCTTGTCCTTGCACTGCACGCAGCCCATGGCGGTA-3′
5	5′-CCGGTACCGCCATGGGCTGCGGCTGCAGCAGCCACCCCATGGC-3′
6	5′-**TCGA**GCCATGGGGTGGCTGCTGCAGCCGCAGCCCATGGCGGTA-3′
7	5′-CCGGTACCGCCATGGGCAGCAGCAAGAGCAAGCCCAAGATGGC-3′
8	5′-**TCGA**GCCATCTTGGGCTTGCTCTTGCTGCTGCCCATGGCGGTA-3′

a the *Age*I single-stranded overhangs are underlined, those of the *Xho*I site are in bold.

**Table 3.** Combinations of oligonucleotides for myr-add mutagenesis.

**Construct**	**Oligonucleotides**
pBC-AktGag-add	1,2
pBC-FynGag-add	3,4
pBC-LckGag-add	5,6
pBC-SrcGag-add	7,8

**Table 4.** Oligonucleotides used for PCR-mediated deletion mutagenesis of the FFV Gag N terminus.

**Primer Name**	**Sequence ^a^**
M1	5′-TGGCTCGAGCGAATCCTCTCCAATTACAG-3′
M2	5′-TGGCTCGAGCGCTCCAATTACAGCAACTG-3′
M3	5′-TGGCTCGAGCGTTACAGCAACTGTATATAAA-′3
M4	5′-TGGCTCGAGCGCAACTGTATATAAATAATGGC-3′
AS-Primer	5′-CCTAGGTTGAATGCAGTTTGT-3′

a *Xho*I restriction site is underlined.
